# Systematic review and meta-analysis of adult multipotent stromal/stem cell treatment for equine tendinopathy and desmopathy

**DOI:** 10.3389/fvets.2026.1758586

**Published:** 2026-03-02

**Authors:** Takashi Taguchi, Mandi J. Lopez, Rita Aoun, Lauren Helber

**Affiliations:** Laboratory for Equine and Comparative Orthopedic Research, Department of Veterinary Clinical Sciences, School of Veterinary Medicine, Louisiana State University, Baton Rouge, LA, United States

**Keywords:** gene, horse, lameness, mechanics, mesenchymal, microstructure, stromal/stem cell, ultrasound

## Abstract

**Introduction:**

Over the last few decades, cell and cell-based therapies emerged as treatment options for equine tendinopathy and desmopathy. The objective of this study was to critically evaluate outcomes following treatment of equine tendinopathy or desmopathy with adult multipotent stromal/stem cells (MSCs).

**Methods:**

The PubMed and Web of Science databases were searched for “equine/horse,” “tendon/tendinopathy/tendonitis/ligament/ligamentopathy/desmopathy/desmitis,” “stem/stromal/mesenchymal/multipotent,” and “cell” from January 2001 to June 2025. Studies were identified according to PRISMA guidelines, and independent reviewers extracted the following information: signalment, lesion location and etiology, treatment, return to soundness or performance, lameness score, ultrasound tissue characterization, and tissue gene expression, composition, mechanical properties, and microstructure. Studies were assessed for risk of bias. A meta-analysis was performed with fixed- or random-effects models and effect size calculated as mean standard deviation or odds ratio, both with 95% confidence intervals, for continuous and dichotomous variables, respectively. Random-effects models were used when heterogeneity was significant.

**Results:**

Seventeen studies met the inclusion criteria for further analysis. Return to soundness or performance, lameness score, ultrasound tissue characterization, and microstructure favored MSC therapy. Neither MSC therapy nor control was favored in tissue gene expression, composition, or mechanics.

**Conclusion:**

Taken together, these findings suggest that adult MSC therapy for equine tendinopathy and desmopathy has a positive effect on clinical outcomes. Randomized controlled trials using standardized cell isolation, preparation, and dosage, as well as outcome measures, are necessary to confirm benefits in tissue mechanics, gene expression, and extracellular matrix recovery.

## Introduction

Tendinopathy and desmopathy comprise a large majority of musculoskeletal injuries in equine athletes (1–3). Injuries resulting from focal accumulation of microtrauma that coalesces into lesions, and weak repair tissue can lead to both acute and chronic pathology (4). Though all horses can experience tendon and ligament pathology, 46% of which includes the superficial digital flexor tendon (SDFT) or suspensory ligament (SL), the predominant injury varies among breeds and activities (5, 6). There is a wide variety of holistic treatment protocols comprised of individual and combined therapies to reduce inflammation, enhance tissue regeneration, and facilitate rehabilitation while minimizing the risk of reinjury (7). Current protocols can include rest, physical treatments such as pressure bandaging and shock wave, laser, and hydro therapies, surgical intervention, medical approaches such as systemic and intralesional therapies, and progressive rehabilitation programs, among others (8). Despite a multitude of treatment options, the overall reinjury rate is as high as 67% within 2 years, and efforts continue to improve both short- and long-term treatment outcomes (9, 10).

Over the last two decades, cell and cell-based therapies, such as stromal/stem cells and platelet-rich plasma (PRP), have emerged as treatment options for equine tendinopathy and desmopathy (11, 12). Intralesional administration of exogenous adult multipotent stromal/stem cells (MSCs) is reported to augment healing in naturally occurring and experimentally induced equine tendon and ligament injuries (13–16). Results are mixed, however, in part due to differences among cell isolates, lesion etiology, individual healing capacity, and cell engraftment (14, 17). Variability among intralesional environments can also influence treatment efficacy since inflammatory mediators reportedly impede progenitor cell differentiation and drive cells to assume unintended phenotypes (18, 19). Comparisons of outcomes among comparable studies are necessary to guide clinical decision-making and research focus.

Existing reviews of cell and cell-based treatments for equine musculoskeletal injuries include treatment with PRP and MSCs separately and together (20–22). A persistent information gap is a comprehensive analysis of both clinical and tissue data from reports of adult MSC therapy for equine ligament and tendon pathology. The aim of this systematic review and meta-analysis of outcomes from MSC administration for experimentally induced and naturally occurring equine tendinopathy and desmopathy was to objectively analyze previous study data together to determine if treatment or control had a more favorable effect on clinical outcomes and tissue characteristics. Risk of bias was subjectively assessed for all studies from which data were extracted. Outcomes assessed were rate of return to performance or soundness, lameness score, ultrasound tissue characterization, and tissue gene expression, composition, mechanical properties, and microstructure. Results of this study represent an objective, contemporary assessment of MSC therapy for equine tendon and ligament damage and provide information for future study design and implementation.

## Materials and methods

### Search strategy

A literature search was carried out using PubMed and Web of Science databases from January 2001 to June 2025. Search strings used for both databases were (equine* OR horse*) AND (tendon OR tendinopathy OR tendonitis OR ligament OR ligamentopathy OR desmopathy OR desmitis) AND (stem OR stromal OR mesenchymal OR multipotent) AND (cell*). Additionally, manual searches were performed in the following journal databases: Veterinary Surgery, Journal of Veterinary Internal Medicine, American Journal of Veterinary Research, Equine Veterinary Education, Equine Veterinary Journal, Journal of Veterinary Emergency Critical Care, and Journal of the American Veterinary Medical Association. Two investigators screened the titles and abstracts of all retrieved studies to remove duplicates and retrieve full texts based on inclusion and exclusion criteria established before study initiation. In cases of disagreement, consensus was reached by the majority based on the decision of a third reviewer. Inclusion criteria were randomized controlled trials or prospective cohort, retrospective cohort, prospective case series, retrospective case series, or prospective longitudinal studies, publication in a peer-reviewed journal, full text accessible through open access, institutional subscription, or interlibrary loan, and availability in the English language. Exclusion criteria consisted of *in vitro* studies, review studies, non-equine species, adult MSC therapy combined with other orthobiologics, and treatment with cells other than adult MSCs.

### Source selection and data extraction

The Population Intervention Comparison Outcome (PICO) rubric in the Preferred Reporting Items for Systematic Reviews and Meta-Analyses (PRISMA) guideline was used to select studies for data analysis (23). Details about investigations included in the meta-analysis consisted of study design, horse signalment (breed, sex, age), limb(s) included, affected structure(s), lesion etiology, MSC treatment, comparator treatment, and follow-up period. The Population was companion or sport horses of any breed, sex, size, or age with naturally occurring or experimentally induced tendinopathy or desmopathy. The studies had to include intra-lesional administration of autologous or allogenic adult MSCs from bone marrow (BMSC), adipose tissue (ASC), tendon tissue (TDPC), or venous (BDMSC) or umbilical cord blood (UCBMSC) as an Intervention. Investigations had to have a Comparator of intralesional administration of serum, saline, the MSC carrier used within the same study, or conventional therapy. Studies also had to include one or more of the following outcomes with at least one specific measure within individual outcomes: rate of return to performance or soundness, lameness score, ultrasound tissue characterization (echogenicity, fiber alignment, lesion or scar size, SDFT cross-sectional area or thickness, vascularity), tissue gene expression (*collagen 1 (Col1)*, *collagen 3 (Col3), cartilage oligomeric matrix protein (COMP), decorin (DCN)*, *matrix metalloproteinase 3 (MMP-3), scleraxis (Scx), tenascin-C (TNC), tenomodulin (TNMD)*), tissue composition (DNA, glycosaminoglycan, total collagen), mechanical properties (elastic *modulus*, maximum or failure stress, stiffness), or microstructure (cellularity, crimp score, collagen type I content, collagen type III content, fiber alignment, fiber structure, inflammatory cell infiltrate, total histology score, vascularity). Quantitative outcome measures were extracted from the records directly or estimated from graphs using software to extract numerical data from images (PlotDigitizer™, Porbital). When the standard error of the mean (SEM) or interquartile range (IR) was reported instead of standard deviation (SD), SD was calculated based on the sample size (SEM) or estimated by dividing by 1.35 (IR) (24). Data from the last assessment point within each study were used for the meta-analysis.

### Study quality and data analysis

Each study was independently assessed by four investigators (TT, RA, LH, MJL) using the Cochrane Collaboration’s risk of bias tool, which contains six categories that are ranked as low, high, or unclear risk. The evaluation criteria for the assessment were: (a) random sequence generation (selection bias), (b) allocation concealment (selection bias), (c) blinding of participants and personnel (performance bias), (d) blinding of outcome assessment (detection bias), (e) incomplete outcome data (attrition bias), and (f) selective reporting (reporting bias). In cases of dissensus, determinations were based on the majority (25). If information was deemed insufficient to assess the risk of bias in a category, an unclear risk of bias was designated.

Data were analyzed with Review Manager software (RevMan 5.4, v9.7.0, The Cochrane Collaboration, London, England). Data in the return to soundness or performance and lameness analyses were allocated to cell subgroups. Individual outcome measures within ultrasound tissue characterization, gene expression, composition, mechanical properties, and microstructure were subdivided into measure – cell type subgroups in multivariate analyses. The mean difference (continuous variables) or the *odds ratio* (dichotomous variables) was determined by the inverse variance method with 95% confidence intervals (CIs). A Cochran Q test was used to test heterogeneity, which was evaluated with *I*^2^ and *χ*^2^ tests. When heterogeneity was significant (*p* < 0.05 or *I*^2^ > 50%), a random effects model was used to estimate pooled outcomes; the CIs and heterogeneity were calculated with the Wald-type method and the restricted maximum-likelihood method, respectively. When heterogeneity was not significant, a fixed effects model was used to determine the OR for categorical variables or the standard mean difference (SMD) for continuous variables with 95% CIs. Weighted SMDs were calculated with the inverse variance method for continuous data among studies using different scales for the same outcome. Results were summarized in forest plots.

## Results

### Study selection and characteristics

**Figure 1:**
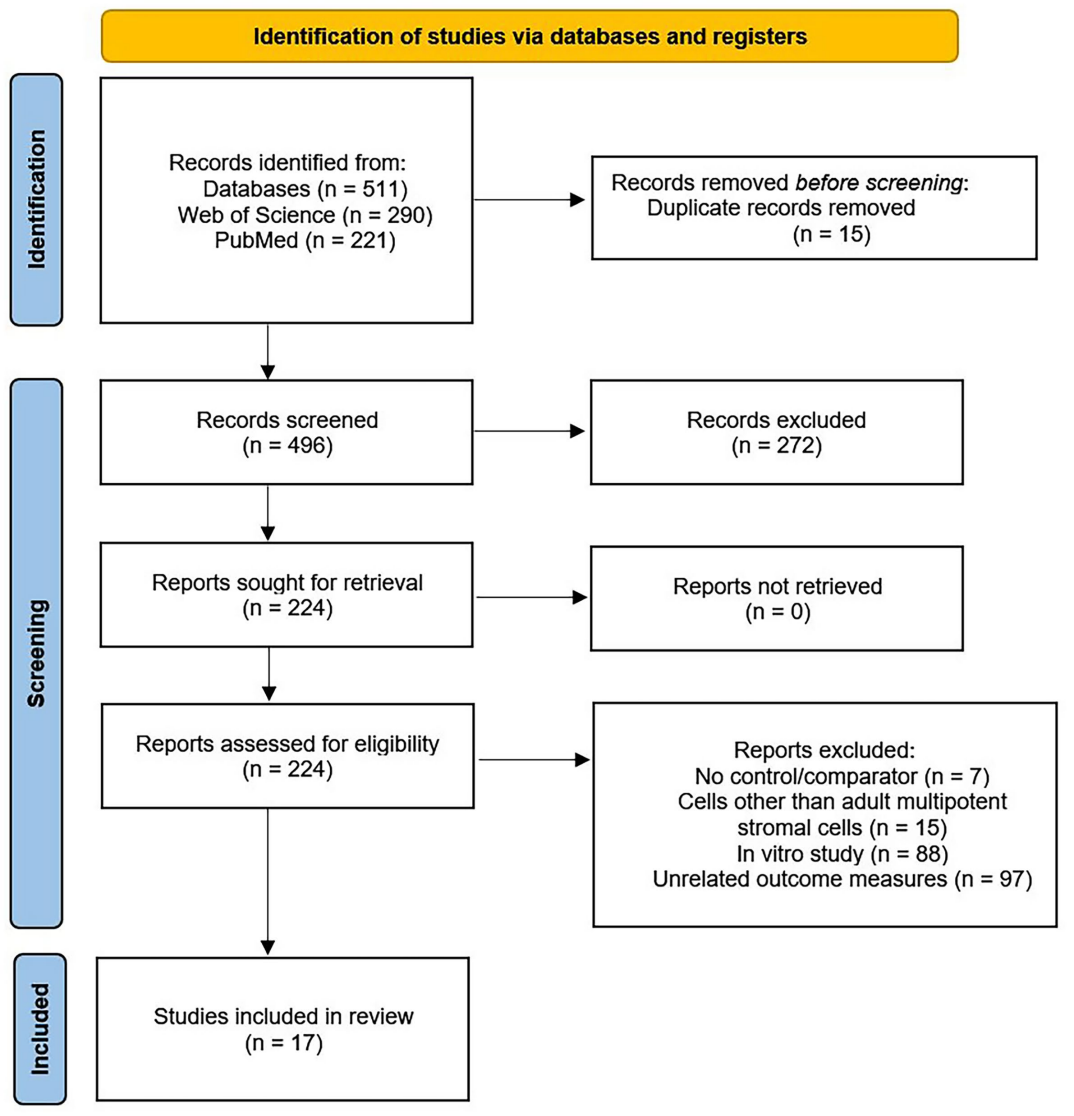
Preferred reporting items of systematic reviews and meta-analyses (PRISMA) flow diagram of the selection process for the systematic literature review and meta-analysis.

A total of 511 publications were identified by the search strategy (Figure 1). After removing duplicate records, 496 records were screened, and 224 were selected for retrieval based on the initial inclusion and exclusion criteria. Of the 224 reports assessed for eligibility, 207 were excluded for the following reasons: no control/comparator (*n* = 7), embryonic stem treatment (*n* = 15), *in vitro* study (*n* = 88), or lacking outcome measures included in the current meta-analysis (*n* = 97). In total, 17 studies were used for the review and meta-analysis: 12 randomized controlled trials (11–14, 16, 26–32), 1 prospective case series (33), 3 retrospective case series (15, 34, 35), and 1 retrospective cohort study (Table 1; Supplementary Table 1) (36).

**Table 1:** Study design, horse breed, sex and age, treated limb(s), treated structure(s), and lesion etiology.

Study	Design	Breed	Sex	Age (years)	Treated Limb (Fore, Hind)	Treated structure	Lesion etiology
Ahrberg et al. (16)	RCT	Standardbred	3 Male 3 Female	Mean: 6 Range: 3–10	Fore, hind	SDFT	Mechanical disruption + collagenase type I
Burk et al. (26)	RCT	*	8 Gelding 6 Mare	Mean: 12.1 Range: 3–25	Fore	SDFT	Natural
Carlier et al. (27)	RCT	Arabian, undefined horse breed, Irish cob, Lusitano, New Forest pony, pinto, undefined pony breed, Pura Raza Espanola, trotter, warmblood	39 Gelding 44 Mare 17 Stallion	Mean ± SD: 12.1 ± 5.0	Fore, hind	SDFT, SL	Natural
Conze et al. (28)	RCT	Warmblood, standardbred	2 Gelding 7 Mare	Mean: 4 Range: 3–6	Fore	SDFT	Mechanical disruption
Crovace et al. (29)	RCT	Standardbred	6 Stallion	Mean: 4	Fore, hind	SDFT	Collagenase type I
DePuydt et al. (30)	RCT	Warmblood	4 Gelding 4 Mare	Range: 3–12	Fore	SDFT	Mechanical disruption
Durgam et al. (37)	RCT	*	8 Undefined	Range: 2–4	Fore	SDFT	Collagenase
Geburek et al. (14)	RCT	Warmblood, trotter	9 Undefined	Mean: 4 Range: 3–6	Fore	SDFT	Mechanical disruption
Marfe et al. (34)	RCS	*	5 Male 1 Female	Range: 10–20	*	SDFT	Natural
Nixon et al. (32)	RCT	*	8 Undefined	Range: 2–6	Fore	SDFT	Collagenase type I
Pacini et al. (35)	RCS	*	20 Male 6 Female	Range: 2–15	*	SDFT	Natural
Rivera et al. (33)	PCS	Holsteiner	10 Undefined	Range: >2	Fore	SDFT	Natural
Romero et al. (12)	RCT	Crossbreed	12 Gelding	Range: 5–8	Fore	SDFT	Mechanical disruption
Salz et al. (36)	Retrospective cohort study	Thoroughbred	113 Gelding 60 Mare 40 Stallion	Range: 3–4	Fore	SDFT	Natural
Schnabel et al. (11)	RCT	*	5 Male 7 Female	Range: 2–5	Fore	SDFT	Collagenase type I
Smith et al. (13)	RCT	Thoroughbred, Thoroughbred cross	13 Gelding	Mean ± SD: 7.8 ± 3.0 Range: 5–15	Fore	SDFT	Natural
Van Loon et al. (15)	RCS	Warmblood	24 Gelding 15 Mare 13 Stallion	Mean ± SD: 9.9 ± 3.5	Fore, hind	SDFT, SL, DDFT, ALDDFT	Natural

*Information not provided. ALDDFT, Accessory ligament of the deep digital flexor tendon; DDFT, Deep digital flexor tendon; PCS, Prospective cohort study; RCS, Retrospective case series; RCT, Randomized controlled trial; SD, Standard deviation; SDFT, Superficial digital flexor tendon; and SL, suspensory ligament.

### Systematic analysis

Warmbloods and thoroughbreds were the most highly represented breeds among the studies, representing close to 50% of the studied populations (Table 1). When the sex of horses was clearly designated, mares and geldings were the most common (12, 13, 26, 28, 30), though stallions were included in at least 4 studies (15, 27, 29, 36). Horse age was 7.0 ± 2.1 years (mean ± SD) with a range of 2 to 25 years. Lesions were limited to the forelimb in the majority of studies (11–14, 26, 28, 30, 32, 33, 36, 37); some studies included the hindlimb as well (15, 16, 27, 29). All studies assessed effects in the SDFT, while one also included the SL (27) and another the SL, deep digital flexor tendon, and accessory ligament of the deep digital flexor tendon (15). Lesions were mechanically induced in four studies (12, 14, 28, 30), initiated chemically (collagenase) in four studies (11, 29, 32, 37), and occurred naturally in eight studies (13, 15, 26, 27, 33–36). In one study, both mechanical disruption and collagenase were used to induce SDFT lesions (16). A total of eight studies compared test and control treatments within the same horse (intrasubject) (11, 12, 14, 16, 28–30, 37) and the rest compared them between horses (intersubject) (Table 2) (13, 15, 26, 27, 32–36). Six studies included the administration of ASCs adipose-derived nucleated cells (14, 16, 26, 28, 32, 33), four BMSCs (11, 13, 29, 35), two both ASCs and BMSCs separately (12, 36), three BDMSCs (27, 30, 34) with (27, 30) and without (34) tenogenic-priming, one TDPCs (37), and one UCBMSCs (15). Autologous cells were used in 12 (11–14, 16, 28, 29, 32–35, 37) and allogenic in four studies (15, 26, 27, 30); autologous BMSCs and allogenic ASCs were tested in one study (36). All studies included intralesional injections, but some studies included multiple injections (2–4, 13, 14, 32), and there was both intralesional and intravenous administration in one study (34). Time between lesion initiation or diagnosis and treatment ranged from 5 to 84 days, and the last assessment point following treatment varied from 8 weeks to 3 years.

**Table 2:** Study comparator location, treatment and comparator, and total evaluation period.

Study	Comparator location (Intrasubject, Intersubject)	Treatment (Tx) and comparator (Ctrl)	Total evaluation period
Ahrberg et al. (16)	Intrasubject	Tx: Autologous ASCs (1×10^7^) + autologous serum (1 mL) Ctrl: Autologous serum (1 mL)	24 wk
Burk et al. (26)	Intersubject	Tx: Allogenic ASCs (5 ×10^6^) + GMP grade horse serum (1 mL)/1 cm^3^ lesion volume Ctrl: GMP-grade horse serum (1 mL)/ cm^3^ lesion volume	18 mo
Carlier et al. (27)	Intersubject	Tx: Tenogenic primed allogenic peripheral blood-derived MSCs Ctrl: 0.9% sodium chloride (1 mL)	112 d
Conze et al. (28)	Intrasubject	Tx: Autologous ASCs (1×10^7^) + inactivated autologous serum (0.5 mL) Ctrl: Autologous serum (0.5 mL)	22 wk
Crovace et al. (29)	Intrasubject	Tx: Autologous BMSCs (~5.5×10^6^) + fibrin glue (~ 4.2 mL) Ctrl: Fibrin glue	21 wk
DePuydt et al. (30)	Intrasubject	Tx: Tenogenic primed allogenic peripheral blood-derived MSCs Ctrl: 0.9% sodium chloride (1 mL)	112 d
Durgam et al. (37)	Intrasubject	Tx: Autologous TDPCs (5.0×10^6^) + PBS (0.15 mL) Ctrl: Saline (0.15 mL)	12 wk
Geburek et al. (14)	Intrasubject	Tx: Autologous ASCs (1×10^7^) + inactivated autologous serum (1 mL) Ctrl: Inactivated autologous serum (1 mL)	24 wk
Marfe et al. (34)	Intersubject	Tx: Autologous CD90^+^ blood-derived stem cells + PBS/gentamicin Ctrl: Conventional therapy	3 yr
Nixon et al. (32)	Intersubject	Tx: Autologous ADNCs (13.83 ± 3.41×10^6^) + PBS (0.6 mL) Ctrl: PBS (0.6 mL)	6 wk
Pacini et al. (35)	Intersubject	Tx: Autologous BMSCs (0.6 to 31.2×10^6^) + autologous serum (1.5 mL) Ctrl: Conventional therapy	~12 mo
Rivera et al. (33)	Intersubject	Tx: Autologous ASCs (0.6×10^6^) + PBS (0.6 mL) Ctrl: Conventional therapy	16 wk
Romero et al. (12)	Intrasubject	Tx: Autologous BMSCs (20×10^6^) or autologous ASCs (20×10^6^) + LRS (7 mL) Ctrl: Lactated Ringer’s solution (7 mL)	45 wk
Salz et al. (36)	Intersubject	Tx: Autologous BMSCs (1×10^7^) or allogenic ASCs (2.1×10^7^) + controlled rehabilitation Ctrl: Controlled rehabilitation	>2 yr
Schnabel et al. (11)	Intrasubject	Tx: Autologous BMSCs (10×10^6^) + PBS (1 mL) Ctrl: PBS (1 mL)	8 wk
Smith et al. (13)	Intersubject	Tx: Autologous BMSCs (10×10^6^) + autologous marrow supernatant (2 mL) Ctrl: Saline (2 mL)	24 wk
Van Loon et al. (15)	Intersubject	Tx: Allogenic UCB-MSCs (2-10×10^6^) Ctrl: Conventional therapy	≥ 6 mo

ASC, Adipose-derived multipotent stromal/stem cell; ADNC, Adipose-derived nucleated cell; ALDDFT, Accessory ligament of the deep digital flexor tendon; BMSC, Bone marrow-derived multipotent stromal/stem cell; CD, Cluster of differentiation; DDFT, Deep digital flexor tendon; GMP, Good manufacturing practices; MSC, Multipotent stromal/stem cell; PBS, Phosphate-buffered saline; SDFT, Superficial digital flexor tendon; SL, Suspensory ligament; TDPC, Tendon-derived progenitor cell; and UCB-MSC, Umbilical cord blood-derived multipotent stromal/stem cell.

**Figure 2:**
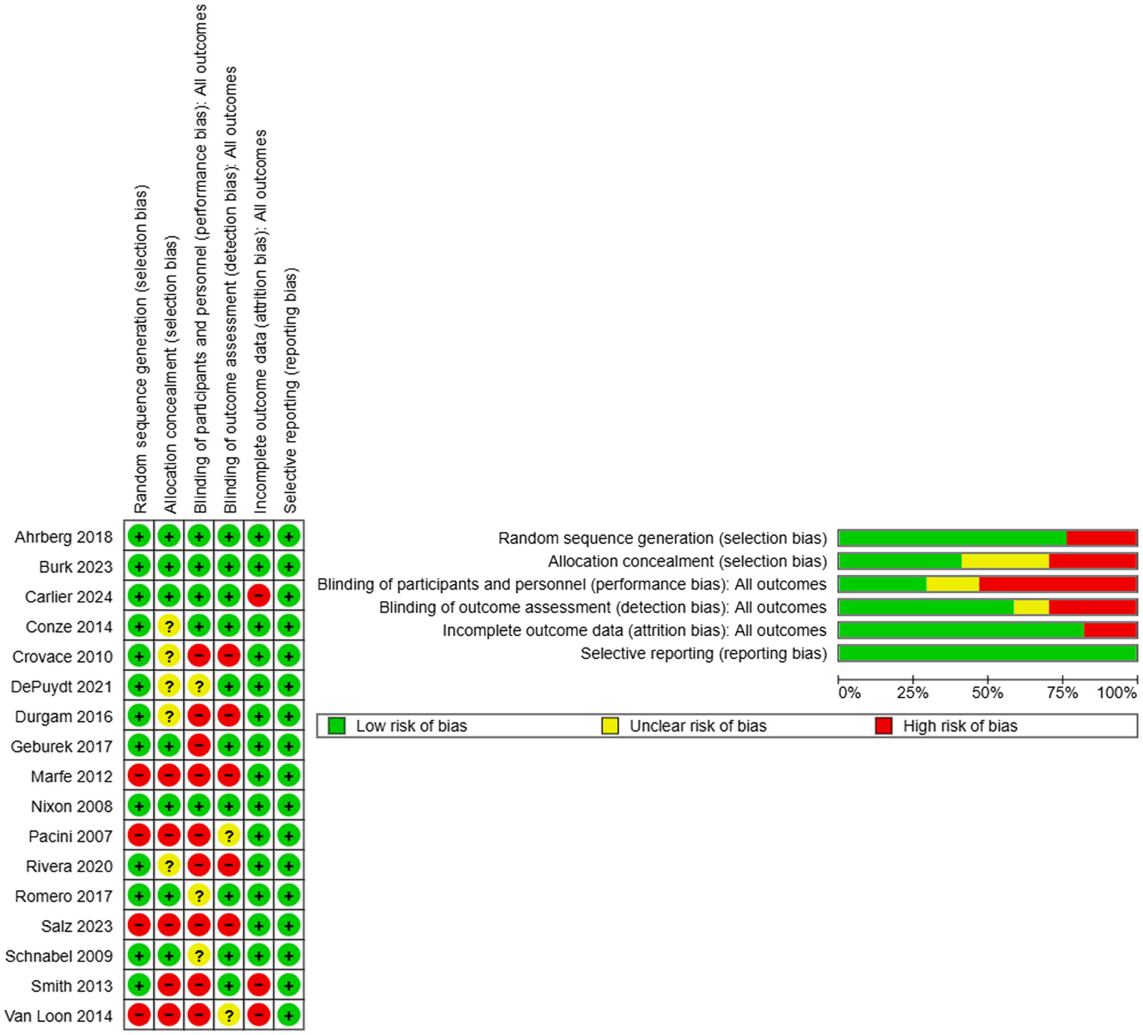
Risk of bias summary (left) and risk of bias graph (right) display judgments about each risk of bias item for all studies included (summary) and each risk of bias item as percentages across all included studies (graph).

A high risk of selection bias was determined in five studies due to no clear indication of randomization (15, 34–36) and/or no concealment of participant allocation to treatment groups (Figure 2) (13, 15, 34–36). Participants and study personnel could not be confirmed to be blinded to treatment in nine studies (13–15, 29, 33–37), and those performing outcome assessments were considered not to be consistently blinded to treatment in five (29, 33, 34, 36, 37). Incomplete outcome data, considered to be a loss of 10% or more participant outcomes for purposes of the evaluation, were determined in three studies (13, 15, 27).

### Meta-analysis

#### Return to soundness or performance

**Figure 3:**
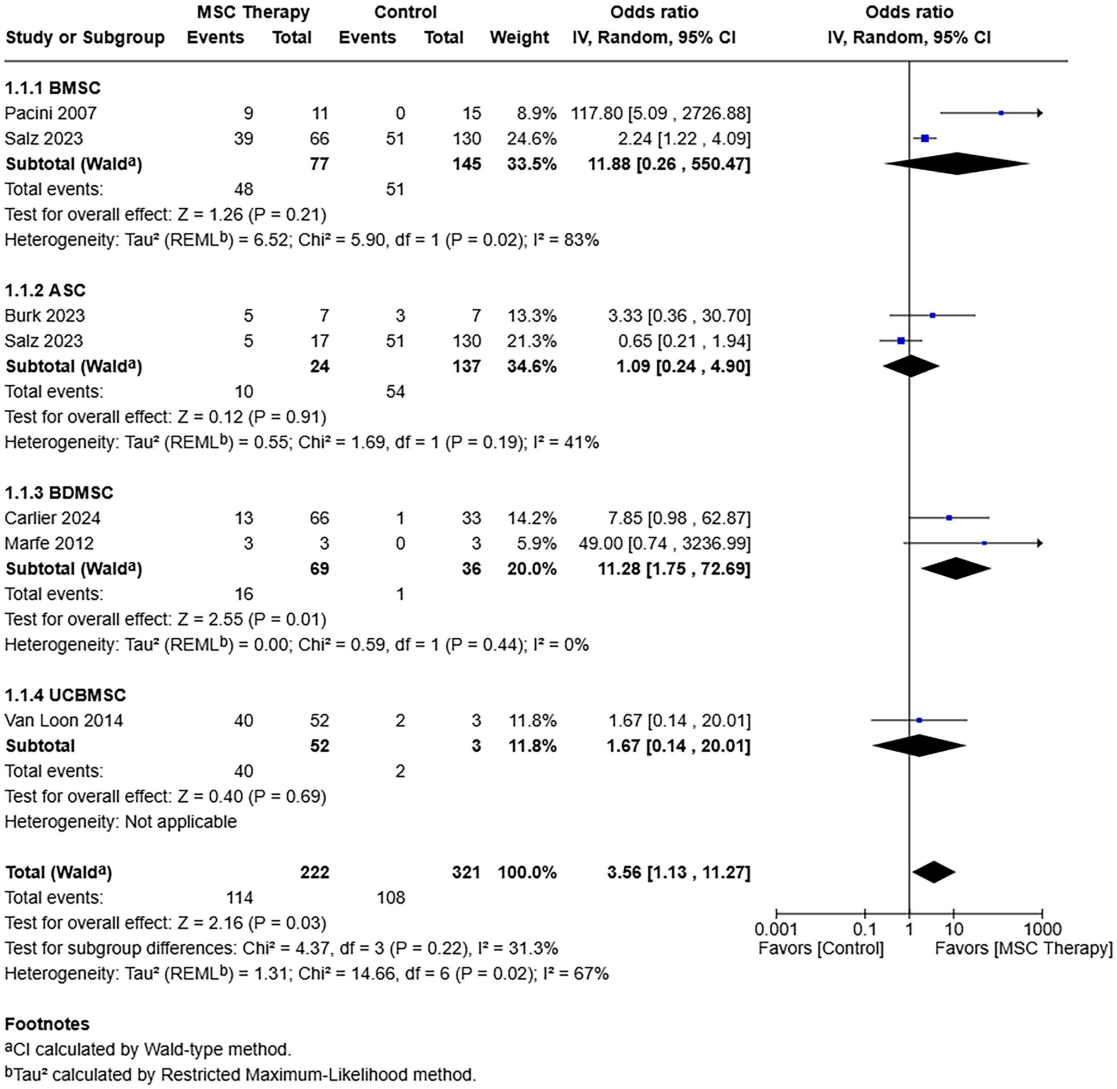
Forest plot of the results of a meta-analysis of the studies indicated to compare return to performance or soundness of adult MSC or control therapy. The results show that the chances of returning to performance or soundness were over 3 times higher with MSC therapy. ASC = Adipose tissue-derived multipotent stromal/stem cell; BDMSC = Blood derived multipotent stromal/stem cell; BMSC = Bone marrow-derived multipotent stromal/stem cell; MSC = Multipotent stromal/stem cell; UCBMSC = Umbilical cord multipotent stromal/stem cell.

Among the six studies that included return to soundness or performance, there were 222 and 321 horses included in the MSC treatment and comparator (control) cohorts, respectively (Figure 3) (15, 26, 27, 34–36). A total of 114 (51.4%) horses in the MSC treatment cohort returned to soundness or performance compared to 108 (33.6%) in the control cohort. A random effects model was applied, given high heterogeneity among studies (*τ*^2^ = 1.31, *χ*^2^ = 14.66, *I*^2^ = 67%, *p =* 0.02). There was no statistically significant heterogeneity in effect sizes among the cell type subgroups, BMSC, ASC, BDMSCs, and UCBMSCs [*𝜒*^2^ = 4.37, (df = 3), *p* = 0.22]. The rate of return to soundness or performance was more favorable following MSCs compared to control therapy [odds ratio = 3.56, 95% CIs (1.13, 11.27), *p* = 0.03].

#### Lameness score

**Figure 4:**
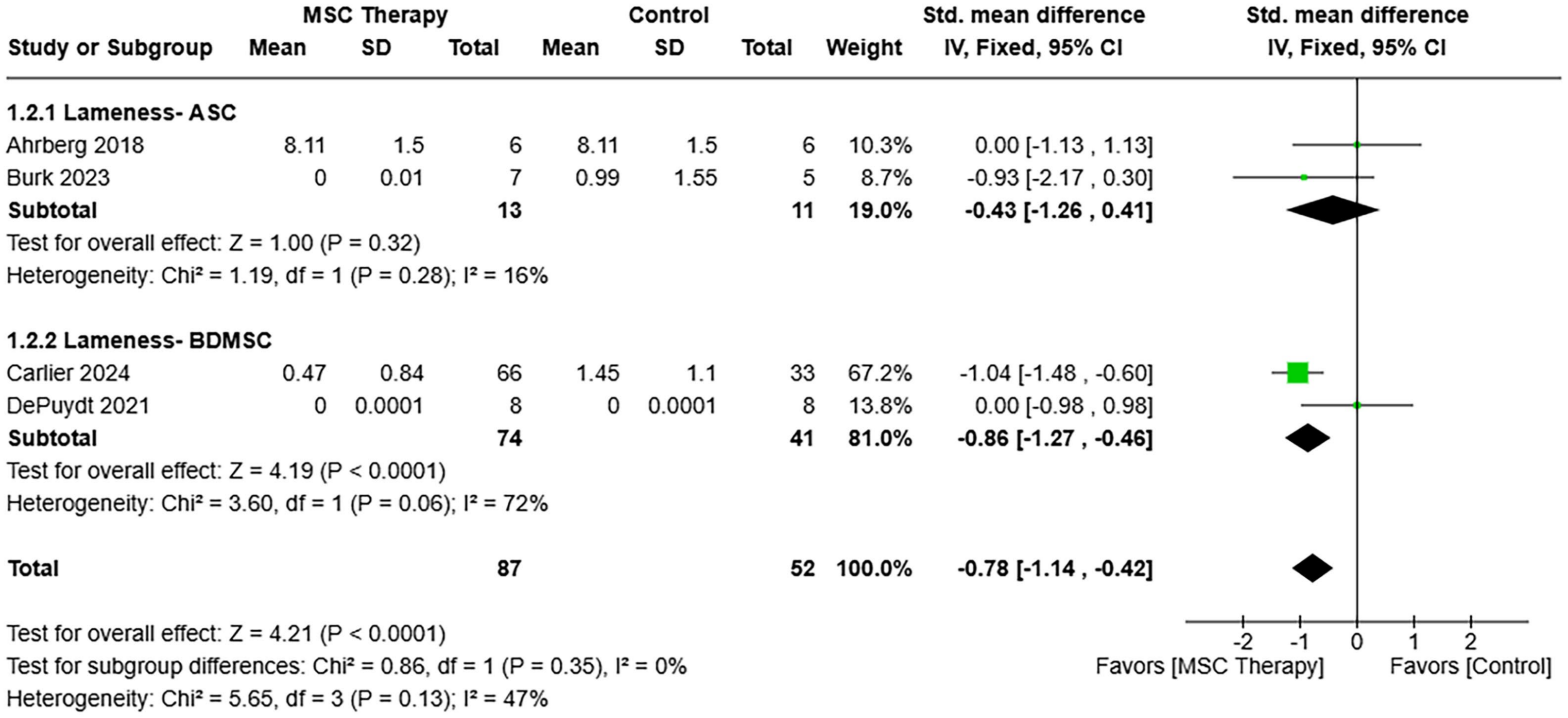
Forest plot of a meta-analysis that shows lameness scores at the last time point included in the studies shown favored over control therapy. Lameness scoring systems included in the analysis had higher scores for greater lameness severity. ASC = Adipose tissue-derived multipotent stromal/stem cell; BDMSC = Blood derived multipotent stromal/stem cell; BMSC = Bone marrow-derived multipotent stromal/stem cell; MSC = Multipotent stromal/stem cell; UCBMSC = Umbilical cord multipotent stromal/stem cell.

Lameness scores from the last evaluation point in the studies included in the assessment favored MSC therapy [SMD = −0.78, 95% CIs (−1.14, −0.42), *p* < 0.0001; Figure 4] (16, 26, 27, 30). A fixed effects model was appropriate for the amount of heterogeneity among studies (*χ*^2^ = 5.65, *I*^2^ = 47%, *p* = 0.13). Heterogeneity in effect size was not significantly different between the cell type subgroups, ASC and BDMSCs [*χ*^2^ = 0.86, (df = 1), *p* = 0.35].

#### Ultrasound tissue characterization

**Figure 5:**
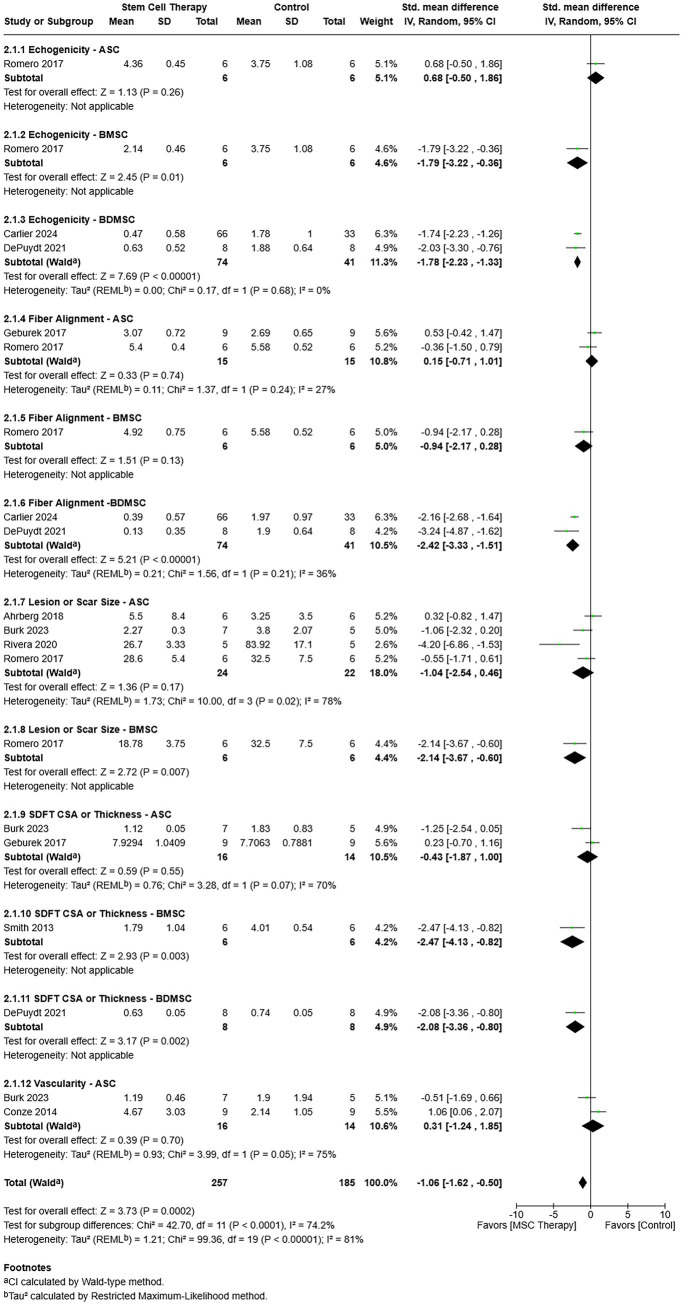
Ultrasound tissue characterization measure-cell subgroups in a forest plot that shows ultrasound outcomes from the last assessment point of studies included in the meta-analysis supported better tissue healing with MSC therapy. Note: Established ultrasound scoring systems utilize scales in which better tissue quality has lower numeric scores. ASC = Adipose tissue-derived multipotent stromal/stem cell; BDMSC = Blood derived multipotent stromal/stem cell; BMSC = Bone marrow-derived multipotent stromal/stem cell; CSA = crossectional area; MSC = Multipotent stromal/stem cell; SDFT = Superficial digital flexor tendon; UCBMSC = Umbilical cord blood multipotent stromal/stem cell.

Ultrasound tissue quality and size were measured using established scales with B-mode ultrasonography. Vascularization was quantified with color Doppler ultrasonography. Ultrasound measure-cell type subgroups for all outcomes included in the review were evaluated together (Figure 5) (12–14, 16, 26–28, 30, 33). A random-effects model was used based on heterogeneity among studies (*τ*^2^ = 1.21, *χ*^2^ = 99.36, *I^2^* = 81%, *p* < 0.00001), and, considered together, MSC therapy was favored over control [SMD = −1.06, 95% CIs (−1.62, −0.50), *p* = 0.0002]. A test for subgroup differences showed statistically significant heterogeneity in effect size among the ultrasound measure-cell type subgroups [*χ*^2^ = 42.70, (df = 11), *p* < 0.0001]. Control was not favored over MSC therapy in any of the subgroups. However, MSC therapy was favored for BMSC echogenicity (*p* = 0.01), BDMSC echogenicity (*p* < 0.00001), BDMSC fiber alignment (*p* < 0.00001), and BMSC lesion or scar size (*p* = 0.007). Furthermore, MSC therapy was favored for BMSC (*p* = 0.003) and BDMSC (*p* = 0.002) SDFT CSA or thickness.

When each outcome measure was assessed independently of the others, one outcome measure, SDFT CSA or thickness, showed an advantage of MSC therapy over control treatment [SMD = −1.29, 95% CIs (−2.52, −0.06), *p* = 0.04]. There was no advantage of MSC therapy or control for echogenicity [SMD = −1.23, 95% CIs (−2.45, −0.00), *p* = 0.05], fiber alignment [SMD = −1.19, 95% CIs (−2.45, 0.07), *p* = 0.06], or lesion or scar size [SMD −1.23, 95% CIs (−2.47, 0.02), *p* = 0.05].

#### Gene expression

**Figure 6:**
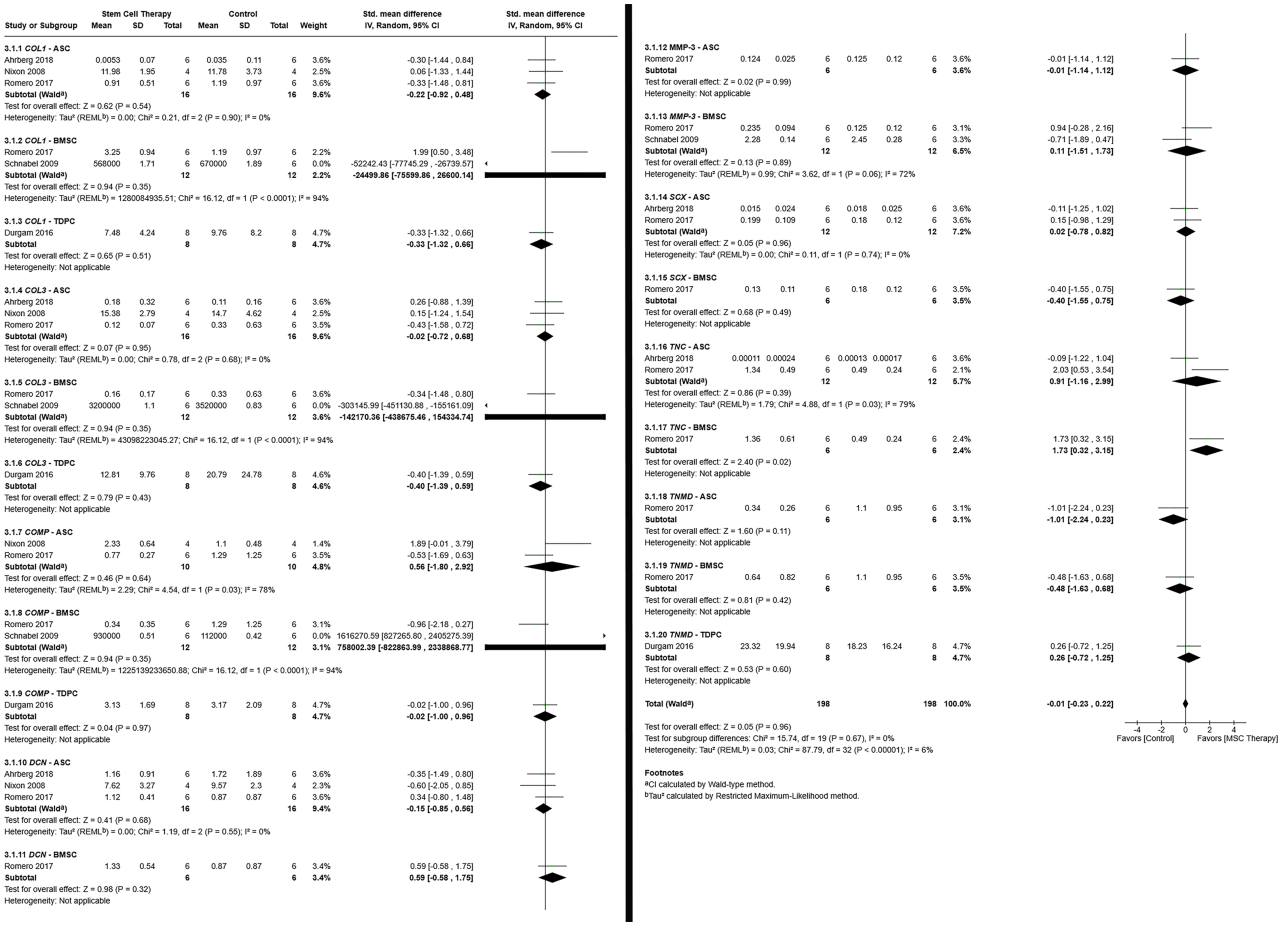
Forest plot of gene-cell type subgroups that demonstrates no difference in tissue gene expression between MSC and control therapies. ASC = Adipose tissue-derived multipotent stromal/stem cell; BMSC = Bone marrow-derived multipotent stromal/stem cell; COL1 = collagen type I; COL3 = Collagen type 3; COMP = Cartilage oligomeric matrix protein; DCN = decorin; MMP-3 = Matrix metalloprotein- 3; MSC = Multipotent stromal/stem cell; SCX = Scleraxis; TDPC = Tendon-derived progenitor cell; TNC = Tenascin-C; TNMD = Tenomodulin.

Data for the expression of eight genes, *Col1* (11, 12, 16, 32, 37), *Col3* (11, 12, 16, 32, 37), *COMP* (11, 12, 32, 37), DCN (12, 16, 32), *MMP-3* (11, 12), *Scx* (12, 16), *TNC* (12, 16), and *TNMD* (12, 37), determined by RT-PCR, were evaluated. The gene expression was quantified as fold change relative to reference genes (2^-ΔCt^) (12, 16), healthy tendon tissue (2^-ΔΔCt^) (37), or total copy number normalized to 18S rRNA expression (11, 32). All gene-cell type subgroups were included in a single analysis (Figure 6). Differences in effect size heterogeneity among subgroups were not significant [*χ*^2^ = 15.74, (df = 19), *p* = 0.67]. Heterogeneity across studies was low but significant (*τ*^2^ = 0.03, *χ*^2^ = 87.79, *I*^2^ = 6%, *p* < 0.00001). Results of a random effects model indicated that differences in gene expression between MSC and control therapies were not significant [SMD = −0.01, 95% CIs (−0.23, 0.22), *p* = 0.96]. The *TNC* – BMSC subgroup was the only subgroup in which MSC therapy was favored (*p* = 0.02), and neither MSC nor control therapy was favored in the remaining subgroups.

#### Composition

**Figure 7:**
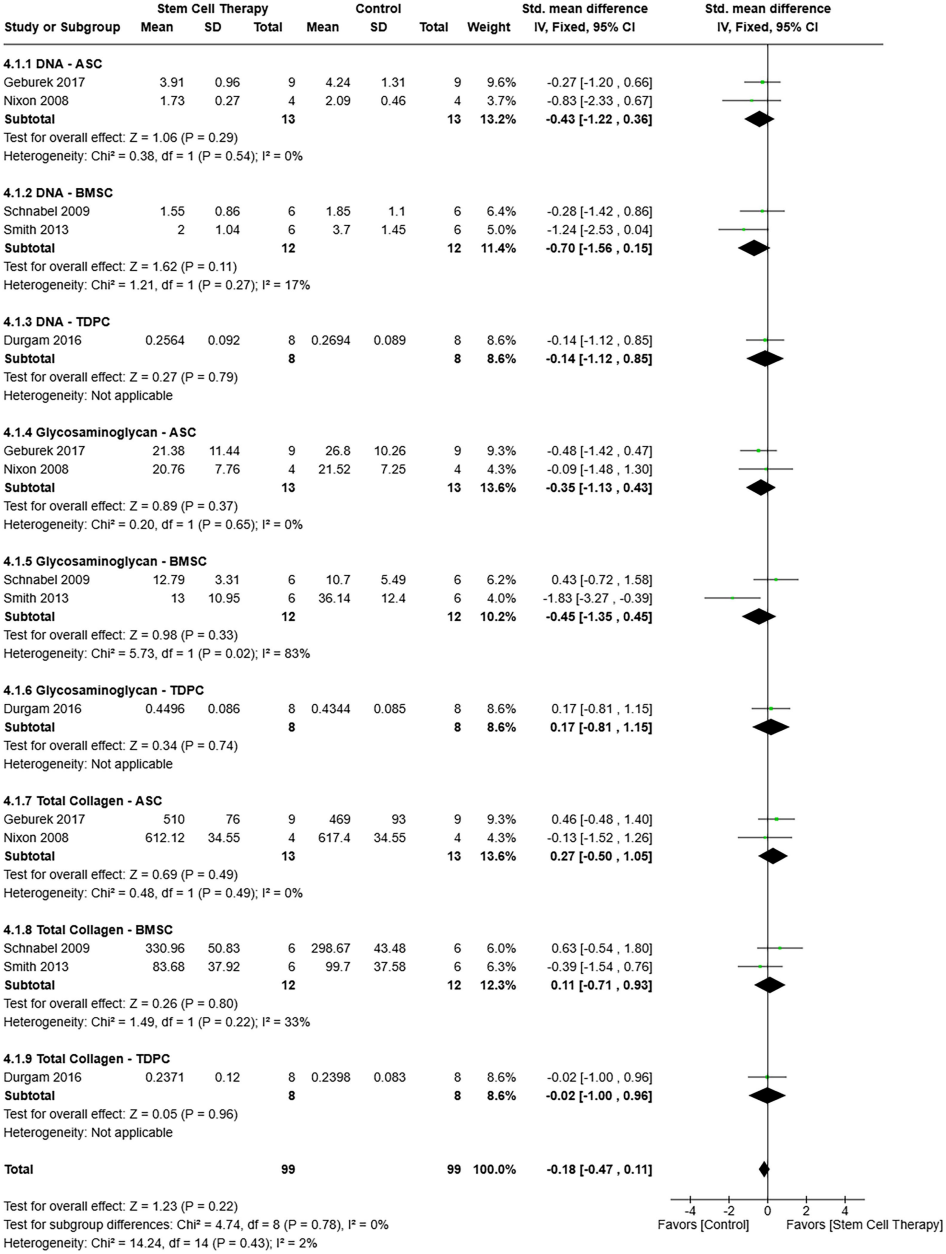
Forest plot with component-cell type subgroups that indicates no difference in tissue composition between MSC and control therapies. ASC = Adipose tissue-derived multipotent stromal/stem cell; BMSC = Bone marrow-derived multipotent stromal/stem cell; MSC = Multipotent stromal/stem cell; TDPC = Tendon-derived progenitor cell.

Among the studies included in the analysis, tissue DNA content was determined with bisbenzimide staining, and glycosaminoglycan content by dimethyl methylene blue dye staining (11, 13, 14, 32, 37). Collagen content was determined indirectly by mass spectrometry (14) or 4-dimethylaminobenzaldehyde quantification of hydroxyproline (13) or directly by picrosirius red staining (11, 32, 37). The values represent the weight of each component relative to the sample dry weight, with all measurements standardized to μg/mg dry weight for the meta-analysis. component-cell type subgroups were included in a single analysis. Differences in effect size heterogeneity between subgroups were not significant [*χ*^2^ = 4.74, (df = 8), *p* = 0.78] (Figure 7). Overall heterogeneity was not significant (*χ*^2^ = 14.24, *I*^2^ = 2%, *p* = 0.43), nor were differences in tissue composition between MSC and control therapies [SMD = −0.18, 95% CIs (−0.47, 0.11), *p* = 0.22] based on a fixed effects model (Figure 7).

#### Mechanical properties

**Figure 8:**
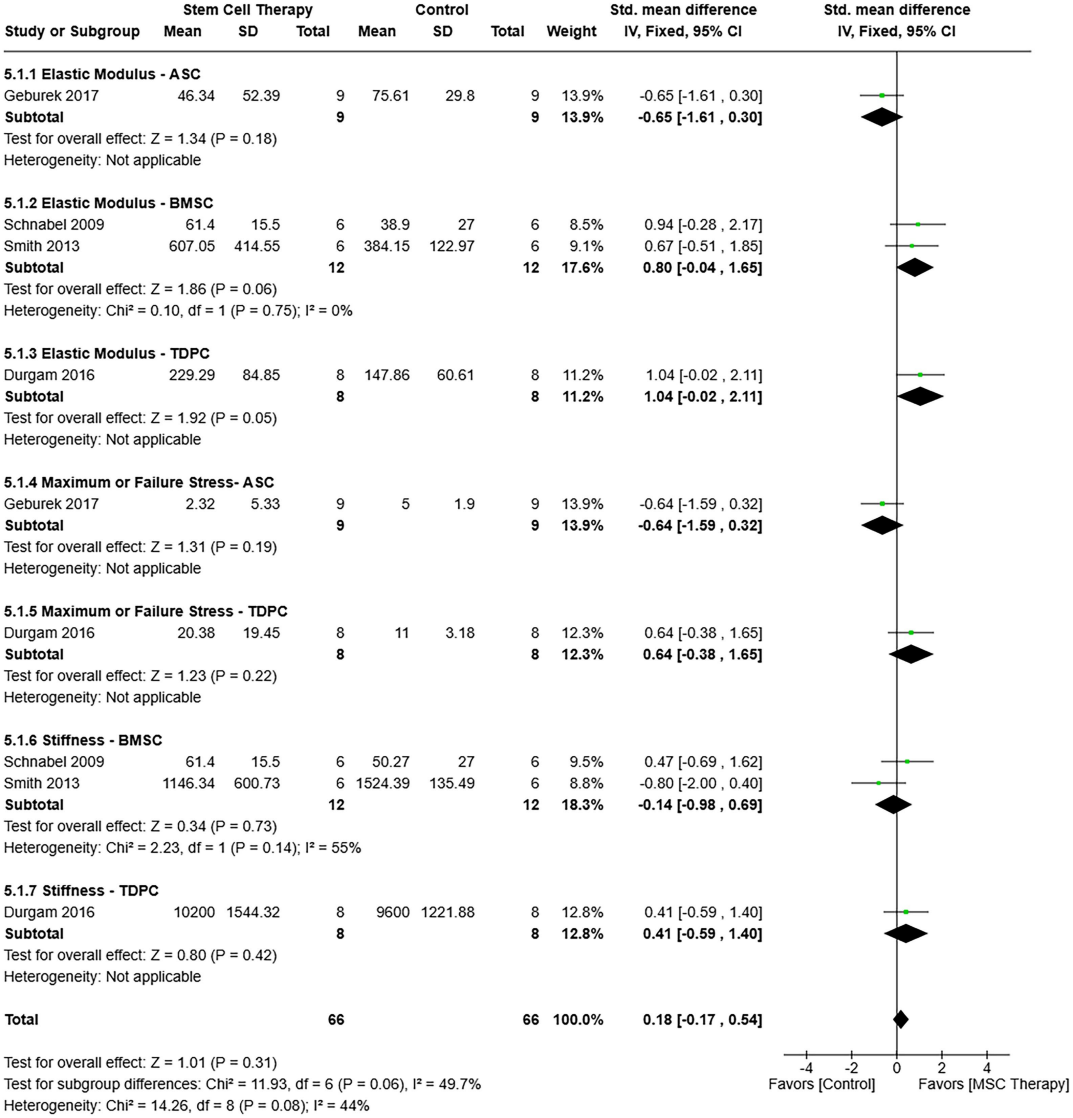
Mechanical property – cell type subgroups within a forest plot in which neither MSC nor control therapy is favored. ASC = Adipose tissue-derived multipotent stromal/stem cell; BMSC = Bone marrow-derived multipotent stromal/stem cell; MSC = Multipotent stromal/stem cell; TDPC = Tendon-derived progenitor cell.

Failure or maximum stress and stiffness, structural properties, and elastic modulus, a material property, were evaluated together in the meta-analysis as property-cell type (Figure 8). The overall heterogeneity across studies was low (*χ*^2^ = 14.26, *I*^2^ = 44%, *p* = 0.08) with a fixed effects model, and differences in subgroup effect size heterogeneity were not significant [χ^2^ = 11.93, (df = 6), *p* = 0.06]. Mechanical property outcomes did not favor MSC or control therapy [SMD = 0.18, 95% CIs (−0.17, 0.54), *p* = 0.31].

#### Microstructure

**Figure 9:**
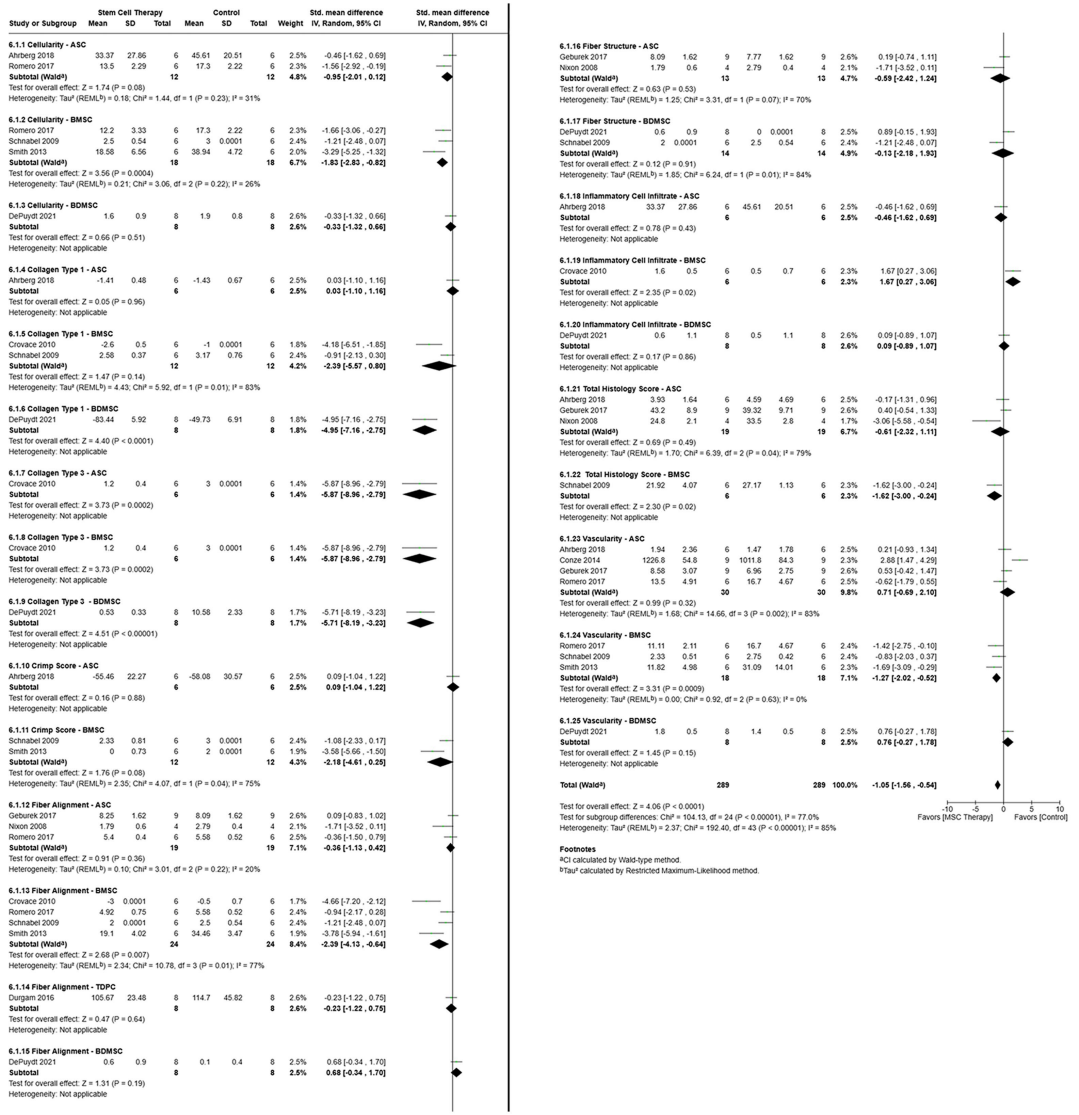
Forest plot with all microstructure-cell subgroups that illustrates more favorable outcomes for MSC therapy compared to control treatments. ASC = Adipose tissue-derived multipotent stromal/stem cell; BDMSC = Blood derived multipotent stromal/stem cell; BMSC = Bone marrow-derived multipotent stromal/stem cell; MSC = Multipotent stromal/stem cell; TDPC = Tendon-derived progenitor cell.

Multiple distinct microstructural outcomes were included in the meta-analysis (Figure 9). They were scored with established rubrics in which a lower score was considered better for healing, with the exceptions noted below. For all but one outcome, COL1, a low score was considered favorable. For consistency, the COL1 scores were entered as negative values, with one exception (11). Cellularity was measured by the percentage of 4’, 6-diamidino-2-phenylindole staining over a region of interest (16) or assigned a score (11–13, 30). Collagen type 1 and 3 content was determined as a percent distribution (30) or scored following immunohistochemical staining (11, 16, 29). Sample crimp analysis was scored with polarized light microscopy after histochemical staining (11, 13, 33); in one study, a higher score was favorable in contrast to the others (16), so the values were entered as negative. Fiber alignment (11–14, 29, 30, 32) and structure (11, 14, 30) and inflammatory cell infiltrate (29, 30) were scored by evaluators following histochemical staining, with one exception (37). In one study, the mean orientation of collagen fibers was determined with second harmonic generation microscopy, and 90° was considered as aligned (37). A composite histology score was provided in four studies (11, 14, 16, 32). Vascularity was scored with established rubrics (11–14, 30), or based on erythrocyte fluorescence (16), or absolute vessel numbers visible after hematoxylin and eosin staining (28).

The overall heterogeneity across studies was high (*τ*^2^ = 2.37, *χ*^2^ = 192.4, *I*^2^ = 85%, *p* < 0.00001), and, based on a random effects model, MSC therapy was favored over control [SMD = −1.05, 95% CIs (−1.56, −0.54), *p* < 0.0001]. Differences in effect size heterogeneity among measure-cell type subgroups were significant [*χ*^2^ = 104.13, (df = 24), *p* < 0.00001]. Control was favored for BMSC inflammatory cell infiltrate (*p* = 0.02). However, MSC therapy was advantageous for BMSC cellularity (*p* = 0.0004), BDMSC COL1 (*p* < 0.0001), ASC (*p* = 0.0002), BMSC (*p* = 0.0002), BDMSC (*p* < 0.00001) COL3, BMSC fiber alignment (*p* = 0.007), BMSC total histology score (*p* = 0.02), and BMSC vascularity (*p* = 0.0009).

When subgroups were analyzed separately with random effects models, MSC therapy was favored for outcomes that included cellularity [SMD = −1.20, 95% CIs (−1.91, −0.49), *p* = 0.0009], COL3 [SMD = −5.80, 95% CIs (−7.44, −4.17), *p* < 0.00001], and fiber alignment [SMD = −1.06, 95% CIs (−2.03, −0.09), *p* = 0.03]. Control therapy was not favored by any of the outcomes.

## Discussion

The major findings of this meta-analysis were that adult MSC treatment of naturally occurring and experimentally induced equine tendon or ligament injuries resulted in an increased rate of return to performance or soundness, lower lameness scores, and better lesion healing based on ultrasound tissue characterization and microstructural examination. While studies varied in design, they all included a negative comparator for purposes of analysis, which was distinct from cell or cell-based product treatments. A comprehensive summary of studies included in the meta-analysis provides essential information on population demographics and study characteristics (Supplementary Table 1). The use of equine adult MSCs from an extensive array of tissue sources to repair damaged tendon and ligament tissue is widely reported; for purposes of this study, however, only studies that fit the *a priori* inclusion and PICO criteria were examined (38). The five tissue sources of adult MSCs included in the meta-analysis were subdivided into individual metrics for ready identification of measure-cell type subgroups within forest plots. Cell type subgroups were examined separately when the effect size heterogeneity was significant among measure-cell type subgroups. Taken together, the information from this review and meta-analysis fills an existing knowledge gap with a detailed analysis of study details and outcomes surrounding adult MSC therapy for equine tendinopathy and desmopathy.

Better return to performance or soundness, reduced lameness score, and improved ultrasound tissue characterization with adult MSC therapy for equine tendinopathy and desmopathy is consistent with previous meta-analyses of cells and cell-based products. Based on a systematic review, treatment with PRP and MSCs alone and together resulted in positive outcomes for equine tendon and ligament pathologies (20). Equine platelet-rich plasma had good short- and medium-term outcomes for tendon and ligament injuries when administered alone, and, when combined with MSCs, it enhanced tissue regeneration and improved long-term outcomes in a systematic review of clinical and experimental studies (21). In a separate systematic review and meta-analysis of reinjury rate and return to performance after treatment of naturally occurring equine tendon and ligament injuries with MSCs and PRP separately and together, the reinjury rate was decreased with cells alone or combined with PRP, but there was no effect on return to performance (22). There was improved ultrasound tissue characterization with MSC therapy in the majority of studies assessed for this review. Administration of PRP was previously reported to improve the ultrasound appearance of equine tendon and ligament lesions in a systematic analysis mentioned above (21). Improved ultrasound appearance was also reported in a systematic analysis of the effects of adult MSC administration on human tendinopathy (39). Taken together, the results of this meta-analysis support macroscopic tissue healing and its associated function.

Improved microstructure with MSC therapy is aligned with the outcomes listed above. In contrast, tissue gene expression, composition, and mechanical properties did not favor either MSCs or control therapy. Gene expression varies temporally with tissue healing, and typically returns to baseline between 8 and 12 weeks after injury (40). In a longitudinal study of healing equine SDFT lesions, *COL1* and *COL3* expression increased immediately after injury, but *COL1* was not significantly different from baseline at any point up to 24 weeks after injury; *COL3* expression was elevated from baseline up to 8 weeks after injury, though it was not different after 24 weeks. Of the five studies that included gene expression (11, 12, 16, 32, 37), only two had data from less than 12 weeks after injury. While early and more robust upregulation of genes associated with tendon healing might be expected with MSC therapy based on microstructural changes, it may be best identified with longitudinal sampling. The same is true for extracellular matrix composition, which might also be expected to parallel microstructure. However, tissue healing is a dynamic, overlapping series of stages that can vary regionally, even within a lesion (41). Notably, COL3 content determined by immunohistochemistry favored MSC therapy. Again, longitudinal sampling would help resolve some of the disparities. Consistent study endpoints could also reduce some variability among studies. The fact that mechanical testing did not support better tissue properties between treatment and control is not surprising, given the number of studies, outcomes, and sample numbers included in the analysis. Individual tissue testing is highly variable, and large sample numbers are often necessary to identify subtle differences among treatment groups (42). Furthermore, the lengthy remodeling process for recovery of native tissue properties can take years (43). The fact that none of the studies evaluated had superior outcomes for either treatment is evidence of the inherent challenges of tendon and ligament mechanical testing.

A 50% high risk of performance bias is a limitation of this meta-analysis. As illustrated in the risk of bias summary, three of 13 randomized controlled trials and five of five non-randomized controlled trial studies (retrospective case series, prospective case series, or retrospective cohort study) were determined to have a high risk of bias in the performance bias category. The inherent design of the non-randomized controlled trial studies makes it challenging or impossible to enact blinding of participants, especially retrospectively. The data from the studies were deemed to be of sufficient value to justify inclusion in the meta-analysis while fully acknowledging performance bias. Future meta-analyses limited to randomized controlled trials are necessary to validate these study findings.

Data used in the meta-analysis were from the last available assessment point in each investigation. However, the length of the total assessment period varied widely among studies, ranging from eight weeks to three years. Lesion etiology was about equally divided between naturally occurring and artificially induced, within which collagenase and mechanical disruption were used to cause injury. Those lesions caused by artificial means tend to have a consistent size and location compared to naturally occurring damage; the lesion and surrounding tissue environments, as well as the tissue response to injury and therapeutic intervention, are undoubtedly distinct between these etiologies (44). Cell dose varied among studies, and the time between injury and treatment was also inconsistent. The cell tissue sources, adipose tissue, bone marrow, tendon, peripheral, and umbilical cord blood, all fit the definition of adult MSCs (45, 46). However, it is well established that tissue source and donor factors impact MSC characteristics, and there is the potential for immune stimulation by autologous or allogenic cells (47). These acknowledged limitations of the data available for meta-analyses should be considered during outcome interpretation.

The number of treatment and control outcomes was unbalanced for return to performance or soundness, ultrasound tissue characterization, and lameness score. Numbers were higher for MSC therapy within lameness score and ultrasound tissue characterization, and higher for control therapy within return to soundness or performance. Unbalanced trials tend to reduce the statistical power of analysis (48). As such, the more favorable outcomes for lameness score and ultrasound tissue characterization might be weakened by the lack of equal cohorts. The multivariate analysis, however, should mitigate some of the effects on ultrasound tissue characterization as described below.

The type and number of available outcomes were distinct among studies. Not all were available as numerical values and were estimated from graphs. For purposes of the meta-analysis, data were subdivided into measure-cell type and evaluated together within a single measure-cell type meta-analysis for most of the major outcome categories. The multivariate approach is advantageous in that the models incorporate within-study correlations among multiple outcomes from the same samples. It also improves precision by including more studies within analyses to account for differing numbers of outcomes from individual studies when some have relatively few compared to others. In all, multivariate analysis increases statistical power and helps to reduce the rate of type I errors. Despite this, cell type subgroups were examined separately for individual measures when the effect size heterogeneity was significant among measure-cell type subgroups. All data used in the meta-analyses can be accessed for independent evaluation (Supplementary Table 1).

### Conclusion and clinical significance

The findings of this meta-analysis indicate that adult MSC treatment of equine tendinopathy and desmopathy has positive effects on return to use and resolution of lameness. Macro- and microstructural healing, evident with ultrasound tissue characterization and histology, corroborate these outcomes. Although assessments of tissue gene expression, composition, and mechanical properties showed no advantage of either adult MSC or control therapy, it is possible that longitudinal sampling is required to identify differences or that these improvements were not detectable within the time periods of the included studies. Additional randomized controlled trials with consistent study designs, treatment protocols, and outcome measures will be essential to advancing the understanding and application of adult MSCs for equine tendinopathy and desmopathy.

## Data Availability

All data used for this study are included in the manuscript and Supplementary Table 1.
