# Supplementary material for: Systematic review and meta-analysis of adult multipotent stromal/stem cell treatment for equine tendinopathy and desmopathy
Source: Front Vet Sci. 2026 Mar 2;13:1758586. doi: 10.3389/fvets.2026.1758586 (PMC12990212; doi:10.3389/fvets.2026.1758586)
Supplement: Supplementary file 1 [file Table_1.pdf]

**Supplemental Table 1.** Study characteristics and outcomes.

| Study Characteristics | Treatment Outcomes | Control Outcomes |
|---|---|---|
| Ahrberg et al, 2018<br>Design: RCT<br>Breed: Standardbred<br>Sex: Male (3), female (3)<br>Age: 6 (3 – 10)<br>Limb: Fore & Hind<br>Lesion location: SDFT<br>Lesion etiology: Mechanical disruption + collagenase<br>Tx: 6 Horses<br>Ctrl: 6 Horses<br>Intrasubject: Yes<br>Tx: Autologous ASCs ($1x10^7$) + autologous serum (1 ml)<br>Ctrl: Autologous serum (1 ml)<br>Post-injury Tx: 3 wk<br>Tx Location: Intralesional<br>Evaluation Period: 24 wk | Clinical<br>Lameness score: 8.11 ± 1.50<br>Ultrasound<br>Lesion CSA: 5.5 ± 8.4%<br>Histology<br>Total score: 3.93 ± 1.64<br>Crimp score: 55.46 ± 22.27%<br>Inflammatory cell infiltrate: 33.37 ± 27.86%<br>Vascularity: 1.94 ± 2.36%<br>Collagen I IHC: 1.41 ± 0.48<br>Gene Expression<br>*COL1A2*: 0.0053 ± 0.07<br>*COL3A1*: 0.18 ± 0.32<br>*Dcn*: 1.16 ± 0.91<br>*Scx*: 0.015 ± 0.024<br>*Tnc*: 0.00011 ± 0.00024 | Clinical<br>Lameness score: 8.11 ± 1.50<br>Ultrasound<br>Lesion CSA: 3.25 ± 3.5%<br>Histology<br>Total score: 4.59 ± 4.69<br>Crimp score: 58.08 ± 30.57%<br>Inflammatory cell infiltrate: 45.61 ± 20.51%<br>Vascularity: 1.47 ± 1.78%<br>Collagen I IHC: 1.43 ± 0.67<br>Gene Expression<br>*COL1A2*: 0.035 ± 0.11<br>*COL3A1*: 0.11 ± 0.16<br>*Dcn*: 1.72 ± 1.89<br>*Scx*: 0.018 ± 0.025<br>*Tnc*: 0.00013 ± 0.00017 |
| Burk et al 2023<br>Design: RCT<br>Breed: *<br>Sex: Male (8), female (6)<br>Age: 12.1 ( 3-25)<br>Limb: Fore<br>Lesion location: SDFT<br>Lesion etiology: Natural<br>Tx: 7<br>Ctrl:7 (5)<br>Intrasubject: No | Clinical<br>Return to soundness: 5/7<br>Lameness: 0 ± 0.01<br>Ultrasound<br>Vascularity: 1.19 ± 0.46%<br>SDFT CSA: 1.12 ± 0.05<br>Lesion size: 2.27 ± 0.30% | Clinical<br>Return to soundness: 3/7<br>Lameness: 0.99 ± 1.55<br>Ultrasound<br>Vascularity1.90 ± 0.1.94%<br>SDFT CSA: 1.83 ± 0.83<br>Lesion size: 3.80 ± 2.07% |

| | | |
|---|---|---|
| Tx: Allogenic ASCs (5 x$10^6$) + GMP grade horse serum (1 ml)/1 $cm^3$ lesion volume<br>Ctrl: GMP-grade horse serum (1 ml)/ $cm^3$ lesion volume<br>Post-injury Tx: <2 m<br>Treatment Location: Intralesional<br>Evaluation Period: 18 m | | |
| Carlier et al 2024<br>Design: RCT<br>Breed: Arabian, undefined horse breed, Irish cob, Lusitano, New Forest pony, pinto, undefined pony breed, Pura Raza Espanola, trotter, warmblood<br>Sex: Gelding (39), mare (44), stallion (17)<br>Age: 12.1 ± 5.0 (SD)<br>Limb: Fore, Hind<br>Lesion location: SDFT, SL<br>Lesion etiology: Natural<br>Tx: 66<br>Ctrl: 34 (33)<br>Intrasubject: No<br>Tx: Tenogenic primed allogenic peripheral blood-derived MSCs (RenuTend®)<br>Ctrl: 0.9% sodium chloride (1 ml)<br>Post-injury Tx: < or ≥ 2 weeks<br>Treatment Location: Intralesional<br>Evaluation Period: 112 days | <u>Clinical</u><br>Return to performance: 13/66<br>Lameness: 0.47 ± 0.84<br><u>Ultrasound</u><br>Fiber alignment: 0.39 ± 0.57<br>Echogenicity: 0.47 ± 0.58 | <u>Clinical</u><br>Return to performance: 1/33<br>Lameness: 1.45 ± 1.10<br><u>Ultrasound</u><br>Fiber alignment: 1.97 ± 0.97<br>Echogenicity: 1.78 ± 1.0 |
| Conze et al, 2014<br>Design: RCT<br>Breed: Warmblood, standardbred<br>Sex: Gelding (2), mare (7)<br>Age: 4 (3 – 6)<br>Limb: Fore | <u>Ultrasound</u><br>Vascularity: 4.67 ± 3.03<br><u>Histology</u><br>Vascularity: 1226.8 ± 54.8 | <u>Ultrasound</u><br>Vascularity: 2.14 ± 1.05<br><u>Histology</u><br>Vascularity: 1011.8 ± 84.3 |

| | | |
|---|---|---|
| Lesion location: SDFT<br>Lesion etiology: Mechanical disruption<br>Tx: 9<br>Ctrl: 9<br>Intrasubject: Yes<br>Tx: Autologous ASCs ($1x10^7$) + Inactivated autologous serum (0.5 ml)<br>Ctrl: Autologous serum (0.5 ml)Tx<br>Post-injury Tx: 14 d<br>Tx Location: Intralesional<br>Follow-up: 22 wk | | |
| Crovace et al, 2010<br>Design: RCT<br>Breed: Standardbred<br>Sex: Stallion (6)<br>Age: 4<br>Limb: Fore, hind<br>Lesion location: SDFT<br>Lesion etiology: Collagenase type I<br>Tx: 6<br>Ctrl: 6<br>Intrasubject: Yes<br>Tx: Autologous BMSCs (~$5.5x10^6$) + fibrin glue (~ 4.2 ml)<br>Ctrl: Fibrin glue<br>Post-injury Tx: 3 wk<br>Tx Location: Intralesional<br>Follow-up: 21 wk | <u>Histology</u><br>Fiber orientation score: 3.0 ± 0.0 (Scored opposite to standard scales).<br>Inflammatory cell infiltrate: 1.6 ± 0.5<br>Collagen I IHC: 2.6 ± 0.5<br>Collagen III IHC: 1.2 ± 0.4 | <u>Histology</u><br>Fiber orientation score: 0.5 ± 0.7<br><br>Inflammatory cell infiltrate: 0.5 ± 0.7<br>Collagen I IHC: 1.0 ± 0.0<br>Collagen III IHC: 3.0 ± 0.0 |
| DePuydt et al, 2021<br>Design: RCT<br>Breed: Warmblood<br>Sex: Gelding (4), mare (4)<br>Age: (3 – 12)<br>Limb: Fore<br>Lesion location: SDFT<br>Lesion etiology: Mechanical disruption | <u>Clinical</u><br>Lameness: 0<br><u>Ultrasound</u><br>SDFT thickness: 0.63 ± 0.05 cm<br>Echogenicity: 0.63 ± 0.52 %<br>Fiber alignment: 0.13 ± 0.35%<br><u>Histology</u><br>Fiber structure: 0.6 ± 0.9 | <u>Clinical</u><br>Lameness: 0<br><u>Ultrasound</u><br>SDFT thickness: 0.74 ± 0.05 cm<br>Echogenicity: 1.88 ± 0.64%<br>Fiber alignment: 1.88 ± 0.64%<br><u>Histology</u><br>Fiber structure: 0 |

| | | |
|---|---|---|
| Tx: 8<br>Ctrl: 8<br>Intrasubject: Yes<br>Tx: Tenogenic primed allogenic peripheral blood-derived MSCs<br>Ctrl: 0.9% sodium chloride (1 ml)<br>Post-injury Tx: 7 d<br>Tx Location: Intralesional<br>Follow-up: 112 d | Fiber alignment: 0.6 ± 0.9<br>Cellularity: 1.6 ± 0.9<br>Vascularity: 1.8 ± 0.5<br>Inflammatory cell infiltrate: 0.6 ± 1.1<br>COLI IHC: 83.44 ± 5.92%<br>COLIII IHC: 0.53 ± 0.33% | Fiber alignment: 0.1 ± 0.4<br>Cellularity: 1.9 ± 0.8<br>Vascularity: 1.4 ± 0.5<br>Inflammatory cell infiltrate: 0.5 ± 1.1<br>COLI IHC: 49.73 ± 6.91%<br>COLIII IHC: 10.58 ± 2.33% |
| Durgam et al, 2016<br>Design: RCT<br>Breed: *<br>Sex: *<br>Age: (2 – 4)<br>Limb: Fore<br>Lesion location: SDFT<br>Lesion etiology: Collagenase<br>Tx: 8<br>Ctrl: 8<br>Intrasubject: Yes<br>Tx: Autologous TDPCs (5.0x10$^{6}$) + PBS (0.15 ml)<br>Ctrl: Saline (0.15 ml)Ctrl: PBS (0.15 ml)<br>Post-injury Tx: 4 wk<br>Tx Location: Intralesional (2 sites)<br>Follow-up: 12 wk | Histology<br>Fiber alignment: 105.67 ± 23.48°<br>Gene Expression<br>*COLI*: 7.48 ± 4.24<br>*COLIII*: 12.81 ± 9.76<br>*COMP*: 3.13 ± 1.69<br>*Tnmd*: 23.32 ± 19.94<br>Composition<br>Collagen: 0.2371 ± 0.12 µg/mg<br>Glycosaminoglycan: 0.4496 ± 0.086 µg/mg<br>DNA: 0.2564 ± 0.092 µg/mg<br>Mechanical Properties<br>Maximum stress: 20.375 ± 19.45 MPa<br>Elastic modulus: 229.29 ± 84.85 MPa<br>Stiffness: 10200 ± 1544.32 N/cm | Histology<br>Fiber alignment: 114.7 ± 45.82°<br>Gene Expression<br>*COLI*: 9.76 ± 8.20<br>*COLIII*: 20.79 ± 24.78<br>*COMP*: 3.17 ± 2.09<br>*Tnmd*: 18.23 ± 16.24<br>Composition<br>Collagen: 0.2398 ± 0.083 µg/mg<br>Glycosaminoglycan: 0.4344 ± 0.085 µg/mg<br>DNA: 0.2694 ± 0.089 µg/mg<br>Mechanical Properties<br>Yield stress: 10 ± 2.47 MPa<br>Maximum stress: 11 ± 3.18 MPa<br>Elastic modulus: 147.86 ± 60.61 MPa<br>Stiffness: 9600 ± 1221.88 N/cm |

| | | |
|---|---|---|
| Geburek et al, 2017<br>Design: RCT<br>Breed: Warmblood, trotter<br>Sex: *<br>Age: 4 (3-6)<br>Limb: Fore<br>Lesion location: SDFT<br>Lesion etiology: Mechanical disruption<br>Tx: 9<br>Ctrl: 9<br>Intrasubject: Yes<br>Tx: Autologous ASCs ($1x10^7$) + inactivated autologous serum (1 ml)<br>Ctrl: Inactivated autologous serum (1 ml)<br>Post-injury Tx: 2 wk<br>Tx Location: Intralesional (2 sites)<br>Follow-up: 24 wk | <u>Ultrasound</u><br>SDFT CSA: 792.94 ± 104.09 $mm^2$<br>Fiber alignment: 3.07 ± 0.72<br><u>Histology</u><br>Total score: 43.20 ± 8.90<br>Fiber alignment: 8.25 ± 1.62<br>Fiber structure: 8.09 ± 1.62<br>Vascularity: 8.58 ± 3.07<br><u>Composition</u><br>Collagen: 510 ± 76 µg/mg<br>Glycosaminoglycan: 21.38 ± 11.44 µg/mg<br>DNA: 3.91 ± 0.96 µg/mg<br>Hydroxyproline: 67 ± 1 µg/mg<br><u>Mechanical Properties</u><br>Failure stress: 2.32 ± 5.33 MPa<br>Elastic modulus: 46.34 ± 52.39 MPa | <u>Ultrasound</u><br>SDFT CSA: 770.63 ± 78.81 $mm^2$<br>Fiber alignment: 2.69 ± 0.65<br><u>Histology</u><br>Total score: 39.32 ± 9.71<br>Fiber alignment: 8.09 ± 1.62<br>Fiber structure: 7.77 ± 1.62<br>Vascularity: 6.96 ± 2.75<br><u>Composition</u><br>Total collagen: 469 ± 93 µg/mg<br>Glycosaminoglycan: 26.80 ± 10.26 µg/mg<br>DNA: 4.24 ± 1.31 µg/mg<br>Hydroxyproline: 62 ± 12 µg/mg<br><u>Mechanical Properties</u><br>Failure stress: 5 ± 1.90 MPa<br>Elastic modulus: 75.61 ± 29.80 MPa |
| Marfe et al, 2012<br>Design: RCS<br>Breed:*<br>Sex: Male (5), female (1)<br>Age: (10 - 20)<br>Limb: *<br>Lesion location: SDFT<br>Lesion etiology: Natural<br>Tx: 3<br>Ctrl: 3<br>Intrasubject: No<br>Tx: Autologous $CD90^+$ blood-derived stem cells + PBS/gentamicin<br>Ctrl: Conventional therapy<br>Post-injury Tx: * | <u>Clinical</u><br>Return to performance: 3 | <u>Clinical</u><br>Return to performance: 0 |

| | | |
|---|---|---|
| Tx Location: Intralesional + intravenous<br>Follow-up: 3 yr | | |
| Nixon et al, 2008<br>Design: RCT<br>Breed: *<br>Sex: *<br>Age: (2-6)<br>Limb: Fore<br>Lesion location: SDFT<br>Lesion etiology: Collagenase type I<br>Tx: 4<br>Ctrl: 4<br>Intrasubject: No<br>Tx: Autologous ADNCs (13.83 ± 3.41 $x10^6$) + PBS (0.6 ml)<br>Ctrl: PBS (0.6 ml)<br>Post-injury Tx: 1 wk<br>Tx Location: Intralesional (3 sites)<br>Follow-up: 6 wk | <u>Histology</u><br>Total score: 24.8 ± 2.1<br>Fiber structure: 1.79 ± 0.6<br><u>Gene Expression</u><br>*COL1*: 11.98 ± 1.95<br>*COL3*: 15.38 ± 2.79<br>*Dcn*: 7.62 ± 3.27<br>*COMP*: 2.33 ± 0.64<br><u>Composition</u><br>Collagen: 612.12 ± 34.55 µg/mg<br>Glycosaminoglycan: 20.76 ± 7.76 µg/mg<br>DNA: 1.73 ± 0.27 µg/mg | <u>Histology</u><br>Total score: 33.5 ± 2.8<br>Fiber structure: 2.79 ± 0.4<br><u>Gene Expression</u><br>*COL1*: 11.78 ± 3.73<br>*COL3*: 14.70 ± 4.62<br>*Dcn*: 9.57 ± 2.30<br>*COMP*: 1.10 ± 0.48<br><u>Composition</u><br>Collagen: 617.40 ± 34.55 µg/mg<br>Glycosaminoglycan: 21.52 ± 7.25 µg/mg<br>DNA: 2.09 ± 0.46 µg/mg |
| Pacini et al, 2007<br>Design: RCS<br>Breed: *<br>Sex: Male (20), female (6)<br>Age: (2 - 15)<br>Limb: *<br>Lesion location: SDFT<br>Lesion etiology: Natural<br>Tx: 11<br>Ctrl: 15<br>Intrasubject: No<br>Tx: Autologous BMSCs (0.6 to 31.2 $x10^6$) + autologous serum (1.5 ml) | <u>Clinical</u><br>Return to performance: 9/11 | <u>Clinical</u><br>Return to performance: 0/15 |

| | | |
|---|---|---|
| Ctrl: Conventional therapy<br>Post-injury tx: *<br>Tx location: Intralesional<br>Follow-up: ~ 12 mo | | |
| Rivera et al, 2020<br>Design: PCS<br>Breed: Holsteiner<br>Sex: *<br>Age: >2<br>Limb: Fore<br>Lesion location: SDFT<br>Lesion etiology: Natural<br>Tx: 5<br>Ctrl: 5<br>Intrasubject: No<br>Tx: Autologous ASCs (0.6 x$10^6$) + PBS (0.6 ml)<br>Ctrl: Conventional therapy<br>Post-injury tx: *<br>Tx location: Intralesional<br>Follow-up: 16 wk | <u>Ultrasound</u><br>Scar length: 26.7 ± 3.33% | <u>Ultrasound</u><br>Scar length: 83.92 ± 17.1% |
| Romero et al, 2017<br>Design: RCT<br>Breed: Crossbreed<br>Sex: Gelding (12)<br>Age: (5 - 8)<br>Limb: Fore<br>Lesion location: SDFT<br>Lesion etiology: Mechanical disruption<br>Tx: 6<br>Ctrl: 6<br>Intrasubject: Yes<br>Tx: Autologous BMSCs (20x$10^6$) or autologous ASCs (20x$10^6$) + LRS (7 ml)<br>Ctrl: LRS (7 ml) | <u>Ultrasound</u><br>ASC: Echogenicity: 4.36 ± 0.45<br>BMSC: Echogenicity: 2.14 ± 0.46<br>ASC: Fiber alignment: 5.4 ± 0.4<br>BMSC: Fiber alignment: 4.92 ± 0.75<br>ASC: Lesion CSA: 28.6 ± 5.4%<br>BMSC: Lesion CSA: 18.78 ± 3.75%<br><u>Histology</u><br>ASC: Cellularity: 13.5 ±2.29<br>BMSC: Cellularity: 12.2 ± 3.33<br>ASC: Fiber alignment: 7.8 ± 3.5<br>BMSC: Fiber alignment: 5.67 ± 2.78<br>ASC: Vascularity: 13.5 ± 4.91<br>BMSC: Vascularity: 11.11 ± 2.11<br><u>Gene Expression</u> | <u>Ultrasound</u><br>Echogenicity: 3.75 ± 1.08<br><br>Fiber alignment: 5.58 ± 0.52<br><br>Lesion CSA: 32.5 ± 7.5%<br><br><u>Histology</u><br>Cellularity: 17.3 ± 2.22<br><br>Fiber alignment: 16.8 ± 4<br><br>Vascularity: 16.7 ± 4.67<br><br><u>Gene Expression</u> |

| | | |
|---|---|---|
| Post-injury tx: 1 wk<br>Tx location: Intralesional<br>Follow-up: 45 wk | ASC: *COLI:* 0.91 ± 0.51<br>BMSC: *COLI*: 3.25 ± 0.94<br>ASC: *COLIII*: 0.12 ± 0.07<br>BMSC: *COLIII*: 0.16 ± 0.17<br>ASC: *COMP*: 0.77±0.27<br>BMSC: *COMP*: 0.34 ± 0.35<br>ASC: *Tnc:* 1.34 ± 0.49<br>BMSC: *Tnc*: 1.36 ± 0.61<br>ASC: *MMP-3*: 0.124 ± 0.025<br>BMSC: *MMP-3*: 0.235 ± 0.094<br>ASC: *Scx*: 0.199± 0.109<br>BMSC: *Scx*: 0.13 ± 0.11<br>ASC: *Tmnd*: 0.34 ± 0.26<br>BMSC: *Tnmd*: 0.64 ± 0.82 | *COLI*: 1.19 ± 0.97<br><br>*COLIII*: 0.33 ± 0.63<br><br>*COMP*: 1.29 ± 1.25<br><br>*Tnc*: 0.49 ± 0.24<br><br>*MMP-3*: 0.125 ± 0.12<br><br>*Scx*: 0.18 ± 0.12<br><br>*Tnmd*: 1.1 ± 0.95 |
| Salz et al 2023<br>Design: Retrospective cohort study<br>Breed: Thoroughbred<br>Sex: Gelding (113), female (60), stallion (40)<br>Age: (3-4)<br>Limb: Fore<br>Lesion location: SDFT<br>Lesion etiology: Natural<br>Tx: BMSC - 66, ASC - 17<br>Ctrl: 130<br>Intrasubject: No<br>Tx: Autologous BMSCs ($1x10^7$) or allogenic ASCs ($2.1\ x10^7$) + controlled rehabilitation<br>Ctrl: Controlled rehabilitation<br>Post injury tx: BMSC ~3 wk, ASC ~7 d<br>Tx location: Intralesional | <u>Clinical</u><br>BMSC Return to performance: 39/66<br>ASC Return to performance: 5/17 | <u>Clinical</u><br>Return to performance: 51/130 |

| | | |
|---|---|---|
| Follow-up: >2 years | | |
| Schnabel et al, 2009<br>Design: RCT<br>Breed: *<br>Sex: Male (5), female (7)<br>Age: (2 - 5)<br>Limb: Fore<br>Lesion Location: SDFT<br>Lesion Creation: Collagenase type I<br>Tx: 6<br>Ctrl: 6<br>Intrasubject: Yes<br>Tx: Autologous BMSCs ($10x10^6$) + PBS (1 ml)<br>Ctrl: PBS (1 ml)<br>Post-injury tx:5 d<br>Tx location: Intralesional<br>Follow-up: 8 wk | Histology<br>Total score: 21.92 ± 4.07<br>Cellularity: 2.5 ± 0.54<br>Vascularity: 2.33 ± 0.51<br>Fiber alignment: 2 ± 0<br>Fiber score: 2 ± 0<br>Crimp: 2.33 ± 0.81<br>COLI IHC: 2.58 ± 0.37<br>Gene Expression<br>*COLI*: $5.68x10^5$ ± 1.71<br>*COLIII*: $3.20x10^6$ ± 1.10<br>*COMP*: $0.93x10^6$ ± 0.51<br>Composition<br>Collagen: 330.96 ± 50.83 µg/mg<br>Glycosaminoglycan: 12.79 ± 3.31 µg/mg<br>DNA: 1.55 ± 0.86 µg/mg<br>Mechanical Properties<br>Stiffness: 61.4 ± 15.5 ksi | Histology<br>Total score: 27.17 ± 1.13<br>Cellularity: 3 ± 0<br>Vascularity: 2.75 ± 0.42<br>Fiber alignment: 2.5 ± 0.54<br>Fiber score: 2.67 ± 0.51<br>Crimp: 3 ± 0<br>COLI IHC: 3.17 ± 0.76<br>Gene Expression<br>*COLI*: 6.7 $x10^5$ ± 1.89<br>*COLIII*: 3.52 $x10^6$ ± 0.83<br>*COMP*: 1.12 $x10^5$ ± 0.42<br>Composition<br>Collagen: 298.67 ± 43.48 µg/mg<br>Glycosaminoglycan: 10.70 ± 5.49 µg/mg<br>DNA: 1.85 ± 1.1 µg/mg<br>Mechanical Properties<br>Stiffness: 50.27 ± 27.0 ksi |
| Smith et al, 2013<br>Design: RCT<br>Breed: Thoroughbred, thoroughbred-cross<br>Sex: Gelding (12)<br>Age: 7.8 ± 3.0 (5 - 15)<br>Limb: Fore<br>Lesion location: SDFT<br>Lesion etiology: Natural<br>Tx: 6<br>Ctrl: 6<br>Intrasubject: No<br>Tx: Autologous BMSCs ($10x10^6$) + autologous marrow supernatant (2 ml)<br>Ctrl: Saline (2 ml)<br>Post-injury tx: 33-75 d<br>Tx location: Intralesional (2 – 4 sites) | Ultrasound<br>SDFT CSA 1.79 ± 1.04 $cm^2$<br>Histology<br>Fiber alignment: 19.10 ± 4.02<br>Cellularity: 18.58 ± 6.56<br>Vascularity: 11.82 ± 4.98<br>Crimp Score: 0 ± 0.73<br>Composition<br>DNA: 2.00 ± 1.04 µg/mg<br>Hydroxyproline: 83.68 ± 37.92 µg/mg<br>Glycosaminoglycan: 13.00 ± 10.95 µg/mg<br>Mechanical Properties<br>Stiffness: 1146.34 ± 600.73 N%<br>Elastic modulus: 607.05 ± 414.55 $N/cm^2$ | Ultrasound<br>SDFT CSA: 4.01 ± 0.54 $cm^2$<br>Histology<br>Fiber alignment: 34.46 ± 3.47<br>Cellularity: 38.94 ± 4.72<br>Vascularity: 31.09 ± 14.01<br>Crimp Score: 2 ± 0<br>Composition<br>DNA: 3.70 ± 1.45 µg/mg<br>Hydroxyproline: 99.70 ± 37.58 µg/mg<br>Glycosaminoglycan: 36.14 ± 12.40 µg/mg<br>Mechanical Properties<br>Stiffness: 1524.39 ± 135.49 N%<br>Elastic modulus: 384.15 ± 122.97 $N/cm^2$ |

| | | |
|---|---|---|
| Follow-up: 24 wk | | |
| Van Loon et al, 2014<br>Design: RCS<br>Breed: Warmblood<br>Sex: Gelding (24), mare (15), stallion (13)<br>Age: 9.9 ± 3.5<br>Limb: Fore, hind<br>Lesion location: SDFT, SL, DDFT, ALDDFT<br>Lesion etiology: Natural<br>Tx: 52<br>Ctrl: 3<br>Intrasubject: No<br>Tx: Allogenic UCBMSCs (2-10x$10^6$)<br>Ctrl: Conventional therapy<br>Post-injury tx: 8-84 days<br>Tx location: Intralesional (≥ 1 site, 1 – 2 times)<br>Follow-up: ≥ 6 mo | Clinical<br>Return to performance: 40/52 | Clinical<br>Return to performance: 2/3 |

Age is presented as mean ± standard deviation, (range), or both, in years. Outcomes included are those from the final study assessment point. Quantified outcomes are presented as mean ± standard deviation. ADNC: adipose-derived nucleated cell; ALDDFT: accessory ligament of the deep digital flexor tendon; ASC: adipose-derived multipotent stromal cell; BMSC: bone marrow-derived multipotent stromal cell; COL1 = collagen type I; COL3 = collagen type 3; COMP = cartilage oligomeric matrix protein; CSA: cross sectional area; Ctrl: control (comparator); DCN = decorin; DDFT: deep digital flexor tendon; MMP-3 = matrix metalloprotein- 3; MSC: multipotent stromal cell; PCS: prospective cohort study; RCS: retrospective cohort study; RTC: randomized controlled trial; SCX = scleraxis; SDFT: superficial digital flexor tendon; SL: suspensory ligament; SML: sesamoidean ligament; Tx = treatment; UCBMSC: Umbilical cord blood multipotent stromal cell; TDPC = tendon-derived progenitor cell; TNC = tenascin-C; TNMD = tenomodulin. * = information not provided.
